# Assessing the Impact of a Virtual Shelter Medicine Rotation on Veterinary Students' Knowledge, Skills, and Attitudes Regarding Access to Veterinary Care

**DOI:** 10.3389/fvets.2021.783233

**Published:** 2021-12-16

**Authors:** Christy L. Hoffman, Terry G. Spencer, Kathleen V. Makolinski

**Affiliations:** ^1^Department of Animal Behavior, Ecology and Conservation, Canisius College, Buffalo, NY, United States; ^2^Maples Center for Forensic Medicine, College of Medicine, University of Florida, Gainesville, FL, United States; ^3^College of Veterinary Medicine, Lincoln Memorial University, Harrogate, TN, United States

**Keywords:** cultural competency, veterinary medicine, access to veterinary care, veterinary student, veterinary education, spectrum of veterinary care, human-animal bond

## Abstract

Strong bonds commonly form between companion animals and people of all socio-demographic backgrounds, yet many pet owners face numerous barriers to accessing veterinary care for their companion animals. For example, they may have difficulties paying for care; they may lack veterinary practices in their community; and they may experience language barriers that impede their ability to utilize veterinary services. Various strategies exist that can help veterinarians address the diverse needs of pet owners in their communities, but these techniques are not commonly covered in the veterinary school curriculum. This study explored how including in-depth, purposefully curated information about access to veterinary care issues within a required shelter medicine rotation impacted fourth-year veterinary students' knowledge, skills, and attitudes regarding the problems clients commonly face when seeking access to veterinary care. Students participated either in a control group of a virtual, four-week rotation delivered via Zoom meetings and self-study, or in an experimental group that additionally completed an interactive online learning module. The online module heavily featured issues surrounding access to veterinary care. Irrespective of which version of the rotation students enrolled, their opinions grew more favorable from pretest to post-test regarding the role of not-for-profit veterinary clinics in communities, as did their expectations that veterinarians should provide affordable treatment options. Additionally, students in the experimental group demonstrated from pretest to post-test increased awareness of the potential for implicit bias toward pet owners within veterinary practice and showed a reduction in their tendency to be judgmental of veterinary clients. By the end of the study, students in the experimental group also expressed greater confidence in their ability to offer incremental care treatment options to veterinary clients. These findings suggest that providing content that focuses on increasing access to veterinary care enhances students' awareness of the need to offer a variety of treatment and payment options to clients. Findings from this study can inform curriculum design in veterinary schools and continuing education programs for veterinary professionals.

## Introduction

People of all socio-demographic backgrounds keep and care for pets, and the strong attachment bonds that commonly form between humans and pets do so irrespective of an owner's income (1, 2). Pet owners with higher incomes and cash liquidity have easier access to veterinary care compared to pet owners with lower incomes and/or less cash liquidity (3). Indeed, individuals in the latter category face numerous challenges to meeting their pets' needs for veterinary care (4, 5). Potential obstacles include the cost of care, accessibility of care, veterinary-client communication difficulties, cultural or language barriers, and a lack of client education (6). Additionally, social determinants of health that influence the delivery of human medical care (e.g., transportation, housing, internet access) also influence the delivery of veterinary care (7). Challenges associated with accessing veterinary care create burdens not only for pet owners and their pets but also for veterinary care providers, who struggle to treat animals effectively when owners lack the resources necessary for treatment.

When preventative veterinary care (e.g., vaccinations and anti-parasitic agents) is not widely available and accessible, viral, bacterial, parasitic, and vector-borne diseases may increase in companion animal populations and compromise human health through the spread of zoonotic disease (8). A recent, intervention-based study demonstrated that by making preventative veterinary care available to low-income pet owners, veterinary visits increased both for wellness and for disease/injury, and monthly heartworm preventative use and vaccination rates increased (9). As pet illness is a common reason pets are relinquished to shelters, often specifically for the purpose of euthanasia (10), veterinary interactions that succeed at educating clients about routine vet care and preventing, or detecting and treating, pets' medical conditions may have an enormous impact on companion animal welfare.

Caring for a sick pet is often a stressful experience for owners, as it can be emotionally draining, time consuming, and costly (11). Additionally, many pet owners live in communities that are not served adequately by veterinary services. Such locations where pet owners have very limited access to veterinary care are referred to as “care deserts” (12) or “veterinary deserts.” While some of these underserved communities are in rural locations where there is a recognized shortage of veterinarians, many underserved communities are located in urban and suburban socio-economically depressed neighborhoods where veterinarians choose not to locate clinics due to financial pressures (6).

Regardless of their clinic's location, veterinarians routinely encounter clients who are struggling to meet their pets' healthcare needs. A survey of over 1,000 small animal practitioners in the United States and Canada indicated that 57% of veterinarians believe that owners' economic limitations affect the care that they are able to provide at least once per day (13). Furthermore, a 2018 survey of over 700 veterinarians in the United States found that 55% perceive the problem of underserved pet populations to be severe (3). The 2018 survey also indicated that veterinarians hold a broad range of views regarding who should own a pet and whether society bears any responsibility for caring for vulnerable people and their pets. For instance, nearly half of participants in that study believed poor people and their pets should be provided with a safety net, yet more than a quarter disagreed with that sentiment. Some participants indicated that changes to the veterinary curriculum could better equip veterinarians with the skills needed to offer effective, lower-cost treatment options (3).

As one of the key stressors veterinarians face is difficult relationships with clients (14) and many low-income individuals have a general distrust of healthcare providers (15), expanding veterinarians' exposure to cultural competency training has the potential to improve the well-being of pets, human clients, and veterinary staff. Cultural competency entails using various interventions in an effort to enhance how effective and accessible services are for individuals from diverse backgrounds (16). Indeed, the North American Veterinary Medical Education Consortium has highlighted diversity and multicultural awareness as a core competency that should be incorporated into veterinary education (17). Such training is not widely implemented within North American veterinary schools; however, integrating cultural competency training into the Australian veterinary curriculum positively affected veterinary students' behaviors and attitudes (18).

Given that obstacles impeding access to veterinary care affect pets, owners, and the veterinary community, there is a need not only for cultural competency training but also for identifying a variety of strategies that enhance access to veterinary care for poor, underserved, and diverse populations. Importantly, such methods must be feasible for staff and clients, favorable to the patient, and financially sustainable for veterinary practices. Veterinary practices have limited ability to offer free or discounted care because doing so can be detrimental to practice sustainability. However, they can offer hospital-based payment plans and partner with third-party services, such as Scratchpay and VetBilling, to provide alternative payment methods for their struggling clients (13).

Another approach is for veterinary practices to encourage the utilization of incremental care and/or spectrum-of-care treatment options. Incremental care is a strategy for delivering care progressively over time using a case-management approach (3). Spectrum of care is a related strategy for providing a variety of care options that might have good outcomes but differing costs and intensity of diagnostics or treatment plans (19). Incremental care and spectrum of care options offer alternatives to doing nothing other than providing the highest level (or gold standard) of treatment. An example of incremental care is the conservative management of a fracture in the distal extremity of select patients with a splint or cast and analgesics at an initial visit. This allows for radiographs, assessment of healing, and reassessment of the treatment plan at a follow-up visit. As another example of incremental care, researchers at Colorado State University have demonstrated that an outpatient treatment protocol for select puppies diagnosed with parvovirus may be a reasonable and less costly alternative to inpatient hospitalization (20). A spectrum of care treatment option might be to manage a feline obstruction case by performing a perineal urethrostomy earlier in the disease course rather than conservatively managing the problem with multiple costly emergency visits (21). Another spectrum of care treatment option is the timely referral of pyometra cases to spay-neuter clinics rather than performing emergency surgery in full-service clinics (22). Such examples allow veterinarians to provide quality care while minimizing expenses for clients. These efforts can thereby strengthen relationships between veterinary team members and the clients they serve.

Currently, most veterinary education programs in the United States emphasize “gold standard” care delivery options over spectrum of care or incremental care treatment options. This is partly because veterinary educators often work in tertiary-care, referral facilities with boarded veterinary specialists rather than in general veterinary practices (3). As a result, veterinary school training at many institutions currently underrepresents the challenges that general practitioners may face and the strategies veterinarians can implement to address these challenges.

The veterinary curriculum is already extensive, yet increasing veterinary students' exposure to cultural competency training, the challenges pet owners face when trying to access veterinary care, alternative payment plans, and incremental or spectrum of care strategies has the potential to improve the lives of pets, their owners, and the veterinary community. Therefore, the goal of this project was to determine how incorporating an interactive, online educational module on issues surrounding access to veterinary care into a virtual shelter medicine rotation would impact veterinary student knowledge, skills, and attitudes toward access to veterinary care issues.

## Methods

### Participants

Fourth year veterinary students at Lincoln Memorial University's College of Veterinary Medicine (LMU CVM) participated in this study during their “Small Animal Primary Care and Shelter Medicine” rotation during the summer of 2020. This rotation, which LMU CVM offers multiple times each year, is required of all veterinary students at this institution, and during the rotation, the students typically spend four weeks onsite at an animal shelter. Due to the novel coronavirus (SARS-CoV-2), which causes coronavirus disease 2019 (COVID-19), students enrolled in the summer 2020 rotations completed their entire rotation virtually. The rotations that occurred after summer 2020 included some in-person components for students. Consequently, data for this study were only collected from the 51 students who were part of the virtual rotations during summer 2020. Whether students were in the experimental or control condition was determined by the rotation to which they were assigned. All students in the first rotation that was part of this study were in the control condition, and all students in the second rotation were in the experimental condition. As students were assigned to their rotation before the study team determined which rotation would be the experimental condition and which would be the control condition, students' assignments were not impacted by the study design.

This study received Institutional Review Board approval from Lincoln Memorial University (IRB #919) and the University of Florida (UF IRB #202001847). Student completion of study-related surveys was optional, and students completed an informed consent document prior to completing the study's pretest survey.

### Measures and Procedures

Students in the control condition were presented with a variety of resources (synchronous learning sessions, webinars, peer reviewed research, program websites, written information, and a video) to improve their understanding of barriers that may prevent clients from seeking veterinary care. The rotation also briefly introduced incremental care treatment strategies, safety net programs, third party payment options for veterinary services, and the role of private practitioners in preventing animal surrender to shelters. Importantly, the control condition did address these issues related to access to veterinary care because such issues naturally arise in a rotation focused on shelter medicine. Therefore, it would have been a disservice to the veterinary students had these topics not been covered. Table 1 identifies the objectives of the shelter medicine rotation.

**Table 1:** Student Learning Objectives (SLOs) associated with the virtual shelter medicine rotation and experienced by all students regardless of their assigned study condition.

**SLOs common to control and experimental groups**	**Knowledge**	**Attitudes/beliefs**	**Skills**
Demonstrate clinical skills using provided performance opportunities.	X		X
Create and maintain accurate medical records for simulated patients.	X		X
Utilize appropriate communication and professional skills during all interactions with others.	X		X
Assess the unique challenges faced by animal shelter and rescue organizations.	X	X	
Demonstrate understanding of relevant veterinary guidelines.	X		
Know the veterinarians' role in preventing the surrender of animals to shelters or rescue groups.	X	X	
Know about Access to Veterinary Care issues and options for clients who have difficulty paying for veterinary care.	X		
Know evidence-based strategies to provide incremental veterinary care.	X		X

In addition to completing a virtual rotation that included the same resources as presented to students in the control condition, students in the experimental condition completed an asynchronous, online learning module that covered in detail the objectives described in Table 2. Students in the experimental condition were presented with a variety of resources (required readings, recorded lectures, and videos) as well as interactive discussions and assignments where they designed incremental treatment care plans and proposed treatment options that fit within patients' budgets. In addition, students in the experimental condition practiced identifying examples of implicit bias, investigated the economic factors on which means testing is based, researched options in their local communities for both for-profit and not-for-profit veterinary care, and considered how social determinants of health impact a client's ability to access veterinary care. The online module did not explicitly introduce spectrum of care but did discuss how veterinary medicine allows for a variety of standards of care that are acceptable practices in different communities and practice settings.

**Table 2:** SLOs associated with the supplemental module and experienced only by students assigned to the experimental condition.

**SLOs unique to experimental group**	**Knowledge**	**Attitudes/beliefs**	**Skills**
Compare major differences and similarities between for-profit, not-for-profit, and municipal governmental veterinary business models.	X	X	
Know how different types of veterinary practices view the issue of access to veterinary care.	X		
Explain how different veterinary business models operating in the same location might affect one another.	X	X	
Recognize the effects that competition and collaboration can have on access to veterinary care.	X	X	
Define “veterinary deserts.”	X		
Define “social determinants of health.”	X		
Recognize how social determinants of health can affect the human-animal bond.	X		
Recognize instances of implicit bias in the practice of veterinary medicine.	X	X	X
Recognize why cultural competence is important for the practice of veterinary medicine.	X	X	
Recognize the range of treatment and financial options available when practicing the veterinary standard-of-care.	X		
Create a treatment plan that allows for incremental care.	X		X
Appreciate the role of low-cost, reduced-cost, and pro-bono veterinary practices in serving the needs of the under-served.	X	X	

Study participants in both the control and experimental groups completed a survey measure at the beginning and end of their shelter medicine rotation. Some of the survey questions were derived from the 2018 CARE Veterinary Service Providers Survey (3), and others were developed specifically for the purposes of the current study. Survey questions were drafted and modified based on feedback received from the rotation instructor (KVM) and the online learning module developer (TGS). Due to time constraints, the survey was not formally piloted; however, an additional veterinary educator with expertise on the topic of access to veterinary care in underserved communities reviewed the survey questions and offered feedback that was incorporated into the survey.

Each study participant created a unique identification code that they entered at the start of both the pretest and post-test so that their responses remained anonymous but their pretest and post-test data could be matched. For the control condition, the pretest and post-test versions of the survey were nearly identical, except participants only entered answers to demographic questions in the pretest survey. The experimental condition surveys included the questions that comprised the control condition surveys. In addition, participants were asked in the experimental post-test survey how much time they spent on the educational module and whether they completed any of the optional module materials (e.g., suggested readings on the module topics). They also were asked to describe their overall observations about the content and activities that comprised the module and to indicate what more they would like to learn about access to veterinary care.

In all versions of the survey, participants answered numerous closed-ended questions. Unless otherwise noted, answer choices were presented on a 7-point Likert scale, which provided a “neutral” option. The first question asked participants to indicate how knowledgeable they were on the topic of access to veterinary care, with answer choices ranging from extremely incompetent to extremely competent. They also were asked to describe in up to 250 words their knowledge on the topic of access to veterinary care for low-income and underserved populations. Participants then expressed their level of agreement with statements that captured their opinions regarding not-for-profit veterinary practices (e.g., “A not-for-profit veterinary clinic should only be allowed to start up in areas where there are currently no for-profit veterinary clinics”), with answer choices ranging from strongly disagree to strongly agree.

Participants indicated how likely they would be to provide veterinary services in a community that lacked veterinary care (i.e., a veterinary desert) in each of the following scenarios: as a veterinarian working at a for-profit clinic; as a veterinarian working at a not-for-profit clinic; and as a veterinarian working at a municipal shelter. Additionally, they were asked about their willingness to volunteer in a veterinary desert. Participants' answer options for these questions ranged from extremely unlikely to extremely likely. They were also asked to indicate how likely they were to be working at a not-for-profit veterinary clinic during the first five years after graduating from veterinary school.

Participants indicated how much they agreed with statements regarding factors that influence pet attachment and the impacts of pets on human well-being (e.g., “Pets can positively impact their owner's health”). Next, they indicated their level of agreement with statements about the association between pet owners' practices and lifestyles and their relationship with their pet and their right to keep a pet (e.g., “People who keep their pets outdoors do not love their pets very much”; “People who surrender their pet to an animal shelter because the pet is sick or injured should not be able to adopt the same pet once treated and recovered”).

The section that followed evaluated students' abilities to identify examples of implicit bias. Students were presented with twelve statements and had to indicate which statements were examples of implicit bias (e.g., “If the client can afford to drive a BMW, she can afford to spay her cat”). These examples were modeled after—but not identical to—examples of implicit bias highlighted in the online module that students in the experimental condition completed. For analysis purposes, the number of times students correctly identified whether a statement was an example of implicit bias was calculated. As there were twelve questions, a student who answered all questions correctly earned a score of twelve on that measure.

Following the implicit bias questions, participants were asked to rate their level of agreement with statements regarding how veterinarians should engage with low-income clients (e.g., “When a client's financial resources are limited, a veterinarian at a for-profit clinic should be willing to provide some care at a level the client can afford rather than providing no care”). Participants then indicated their level of agreement with statements about the value of collaborations between veterinary services and social services organizations. Next, participants rated their confidence in their ability to treat animals using incremental care and affordable treatment options (e.g., “To diagnose an animal's medical condition without the use of high-tech equipment”) on a slider scale ranging from 0 to 100, with 100 representing the highest level of confidence.

In the last part of the survey, participants provided information regarding their age; gender; history of volunteering at for-profit and not-for-profit veterinary clinics and municipal animal shelters; their experiences taking college-level courses on animal shelters and/or human social services; whether they had participated in a shelter medicine club; and their race and ethnicity.

### Data Analysis

To assess the internal consistency of each measure, Cronbach's alphas were calculated (see Table 3). Responses to eight questions were averaged to develop a composite judgment of clients score (α = 0.85), and scores on four questions were averaged to characterize students' attitudes regarding not-for-profit veterinary clinics (α = 0.82). Responses to three questions were used to characterize participants' opinions regarding how pets affect owner health, stress, and physical activity (α = 0.67), while responses to five questions were averaged to characterize students' expectations regarding whether veterinarians should help clients whose financial resources are limited (α = 0.76). Answers to three questions were averaged to assess students' confidence in their ability to offer incremental care plans (α = 0.90).

**Table 3:** Measures and the questions that comprised them.

	**α**
Judgement of clients (higher score indicative of more judgmental attitude)	0.85
Pet ownership is a privilege and not a right	
People who keep their pets outdoors do not love their pets very much	
People commonly use poverty as an excuse for neglecting their pets	
Some pet owners are more likely than others to face obstacles when seeking veterinary care for their pets (Reverse scored)	
People who surrender their pets to shelters should not be allowed to adopt a pet in the future	
People who surrender their pets to shelters lack compassion	
If a family is not on any form of public assistance (e.g., Supplemental Nutrition Assistance Program, formerly known as Food Stamps), they should be willing and able to pay for the best possible treatment option for their pet	
People who surrender their pet to an animal shelter because the pet is sick or injured should not be able to adopt the same pet once treated and recovered	
Regard for not-for-profit veterinary clinics (higher score indicative of higher regard, meaning all items were reverse-scored)	0.82
Not-for-profit veterinary practices should be required to qualify their clients by income (i.e., perform means testing)	
Not-for-profit veterinary clinics negatively impact revenue for for-profit veterinary clinics that are in the same community	
A not-for-profit veterinary clinic should only be allowed to start up in areas where there are currently no for-profit veterinary clinics	
Not-for-profit veterinary practices should lose their not-for-profit tax status if they do not qualify their clients by income (i.e., perform means testing)	
Effects of pets on health	0.67
Pets can positively impact their owner's health	
Pets can reduce owners' stress levels	
Pets can impact individuals' physical activity levels	
Expectation that veterinarians provide affordable treatment options	0.76
When a client's financial resources are limited, a veterinarian at a for-profit clinic should be willing to provide some care at a level the client can afford rather than providing no care	
There are financially sustainable ways in which for-profit veterinary clinics can treat sick pets that belong to low-income clients	
There are ethically sound ways in which for-profit veterinary clinics can treat sick pets that belong to low-income clients	
Providing access to veterinary care is part of the “social ethic” mandate and therefore the responsibility of those in the veterinary profession	
Providing some care at a level the client can afford (i.e., incremental care) can positively impact an animal's quality of life	
Confidence in ability to provide incremental care	0.90
To diagnose an animal's medical condition without the use of high-tech equipment	
To create effective care plans that utilize alternatives to the best possible treatment options	
To present economically disadvantaged clients with alternative, more affordable treatment options when their pets are ill	

All analyses were conducted using R version 3.6.3 (21). Linear mixed models assessed whether there were main effects of study condition and test timing (i.e., pretest or post-test) on study measures. Statistical models also tested for interactions between study condition and test timing. Participant was treated as a random factor in all models because each participant provided pretest and post-test data. Outcomes that were based on each participant's averaged scores on the measures described in Table 3, or on the total number of implicit bias questions answered correctly, were assessed using a Gaussian distribution unless the distribution of residuals was skewed. In such cases, a median cutpoint was determined and analyses were conducted using a binomial distribution. Each participant's self-reported assessment of how knowledgeable they were on the topic of access to veterinary was comprised of a single question with Likert scale response options. Similarly, individual questions with Likert scale response options assessed how likely participants were to work or volunteer in a veterinary desert in the future under a variety of contexts. In these cases, a median cutpoint was utilized by categorizing each score as being above or below/equal to the median to make the dependent variable binary, and analyses were conducted using a binomial distribution.

TGS reviewed and analyzed participants' responses to four pre- and post-rotation, open-ended survey questions. The four questions are listed below this paragraph. TGS was not an instructor for the rotation and was not privy to student names. She only had access to the anonymized identifiers associated with the students. However, there is some potential for bias, as TGS was the primary author of the online module presented to the experimental group. TGS used a deductive, content analysis approach to identify patterns in the written student responses. The content analysis was managed using NVIVO software. An iterative process was followed, and this involved reading and coding the student responses about their learning experience multiple times in order to categorize the details students reported.

- **Question 1 (Pretest) and Question 2 (Post-test) were asked of those in both groups:** *What do you know regarding the topic of Access to Veterinary Care for low-income and underserved populations?*
- **Question 3 (Post-test, asked of those in the experimental group only):** *Please describe your overall observations about the content and activities that comprised the Access to Veterinary Care module*.
- **Question 4 (Post-test, asked of those in the experimental group only):** *What more would you like to learn about Access to Veterinary Care?*

Student responses to questions 1 and 2 were initially rated according to students' levels of familiarity with the subject of access to veterinary care, as indicated by the quantity and level of details they voluntarily included within their answers (23). That is, a more detailed response was considered to be associated with more familiarity with the subject. Classifications used included minimal familiarity, moderate familiarity, and familiar. In addition, a key word search was used to determine the frequency at which students mentioned within their responses to questions 1–4 specific topics or concepts presented in their learning activities. The topical codes that were identified within the student responses included the following: veterinary deserts, social determinants of health, liquidity, implicit bias, human-animal bond, cost of care, pro-bono-care, pet insurance, pet food pantry, payment plans, incremental-care, grants and subsidies, and communication skills.

## Results

Twenty-five individuals completed the pretest and the post-test surveys as part of the control condition, and 26 completed the pretest and post-test surveys as part of the experimental condition. No Lincoln Memorial University students who were enrolled in the study's shelter medicine rotations during summer 2020 opted out of participating in the study. Participants in the control group ranged in age from 23 years to 31 years (*M* = 26.4, SD = 1.83). Those in the experimental group ranged in age from 24 years to 38 years (*M* = 26.9, SD = 3.05). Twenty-three of the 25 participants in the control group identified as female, and two identified as male. In the experimental group, 23 participants identified as female, and three identified as male. Additional details about the participants and their educational and experiential backgrounds are included in Table 4. On average, those in the experimental condition spent 7.71 h (SD = 2.94, range: 3–15 h) working through the online access to veterinary care module.

**Table 4:** Description of study participants.

	**Control group**	**Experimental group**
*n*	25	26
Number of females	23 (92%)	23 (88%)
**Race**		
African American	0 (0%)	1 (4%)
Asian	0 (0%)	1 (4%)
Biracial	1 (4%)	1 (4%)
White	24 (96%)	22 (85%)
Did not disclose	0 (0%)	1 (4%)
**Ethnicity**		
Of Latino, Hispanic, or Spanish origin	3 (12%)	3 (11%)
Not of Latino, Hispanic, or Spanish origin	22 (88%)	22 (85%)
Did not disclose	0	1 (4%)
**Work/volunteer experience**		
For-profit veterinary clinic	25 (100%)	26 (100%)
Not-for profit veterinary clinic	11 (44%)	4 (15%)
Municipal animal shelter	13 (52%)	13 (50%)
Veterinary clinic that provided free or reduced cost care	17 (68%)	14 (54%)
**Coursework**		
Course on animal shelters	2 (8%)	1 (4%)
Course on human-focused social services	3 (12%)	3 (12%)
Participated in a shelter medicine club	12 (48%)	9 (35%)

At the beginning of the pretest and post-test surveys, participants were asked to rate their knowledge on the topic of access to veterinary care for low-income and underserved populations. There was no effect of condition on how participants rated their knowledge (β = 0.58, SE = 4.19, *p* = 0.89), and the interaction between condition and test was not significant (β = −2.76, SE = 6.01, *p* = 0.65). Participants did indicate their knowledge increased from pretest to post-test, however (β = 24.54, SE = 6.10, *p* < 0.001).

### Ability to Detect Examples of Implicit Bias

Participants in the control condition answered 10.7 (SD = 1.46) of the 12 implicit bias questions correctly during the pretest and 10.6 (SD = 1.66) during the post-test. Those in the experimental condition answered 9.7 (SD = 1.78) correctly in the pretest and 11.6 (SD = 0.99) during the post-test. When a regression was performed using a Gaussian distribution, the residuals were not normally distributed; thus, a binomial logistic regression was performed using the median number correct on the implicit bias assessment as the median cut point. The main effects of study condition and test were not significant (condition: β = −0.70, SE = 0.69, *p* = 0.31; test: β = 0.37, SE = 0.62, *p* = 0.55), but the interaction between condition and test was significant (β = 2.30, SE = 1.00, *p* = 0.02). Individuals in the experimental condition showed an improvement on the implicit bias assessment from pretest to post-test whereas individuals in the control condition did not.

### Judgment of Clients

**Figure 1:**
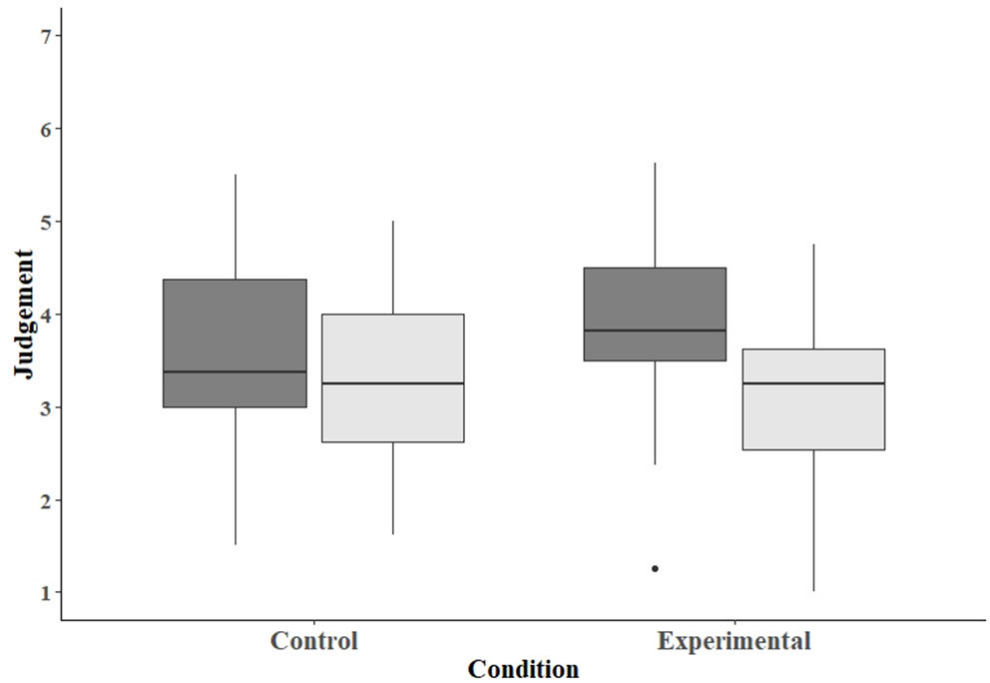
Box plot depicting the scores assessing tendencies of participants in the control and experimental conditions to judge clients. Dark-gray bars represent scores on the pretest, and light-gray bars scores on the post-test.

Participants responded to questions regarding their judgments about clients' behaviors and whether their behaviors should impact their ability to adopt pets in the future. Those in the control condition scored 3.60 (SD = 1.09) on the pretest and 3.28 (SD = 0.97) on the post-test (Figure 1). Those in the experimental condition scored 3.89 (SD = 1.01) on the pretest and 3.02 (SD = 0.95) on the post-test. There was no main effect of condition (β = 0.30, SE = 0.27, *p* = 0.27), but the main effect of test was significant (β = −0.33, SE = 0.13, *p* = 0.01). The interaction between condition and test also was significant (β = −0.53, SE = 0.19, *p* = 0.006). That is, the decrease in judgment scores was greater from pretest to post-test for those in the experimental condition than for those in the control condition.

### Regard for Not-for-Profit Veterinary Clinics

**Figure 2:**
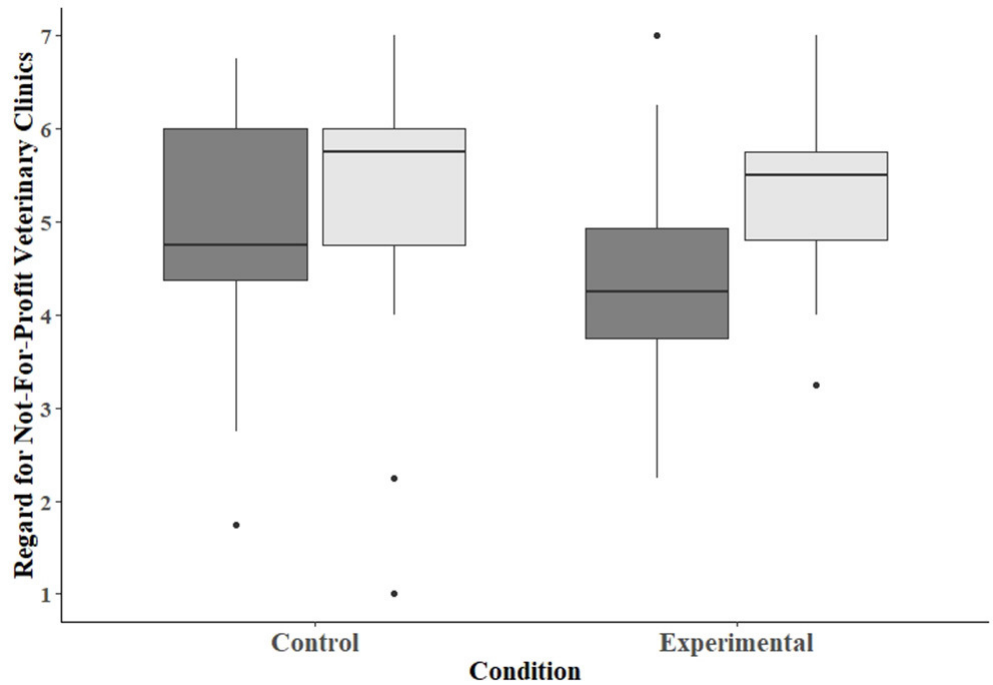
Box plot depicting the scores assessing participants' regard for not-for-profit veterinary clinics. Dark-gray bars represent scores on the pretest, and light-gray bars scores on the post-test.

Regarding participants' opinions about not-for-profit veterinary clinics, the average participant scores for those in the control condition was 4.92 (SD = 1.35) on the pretest and 5.38 (SD = 1.37) on the post-test (Figure 2). For those in the experimental condition, the average score was 4.40 (SD = 1.06) on the pretest and 5.29 (SD = 0.86) on the post-test. The main effect of condition was not significant (β = −0.50, SE = 0.32, *p* = 0.12), but the main effect of test was (β = 0.45, SE = 0.20, *p* = 0.03). The interaction between condition and test was not significant (β = 0.42, SE = 0.28, *p* = 0.13), meaning that regard for not-for-profit veterinary clinics increased significantly from pretest to post-test, regardless of study condition.

### Importance of Social Work Partners

Participants in both conditions, irrespective of whether they were taking the pretest or post-test, indicated they highly valued collaborations between the veterinary community and human social services agencies. Out of a maximum score of 7, with 7 representing the highest possible regard for partnerships between veterinarians and social services agencies, the average scores for participants in the control condition were 6.09 (SD = 1.28) on the pretest and 6.39 (SD = 0.74) on the post-test. The average scores for participants in the experimental condition were 6.43 (SD = 0.71) on the pretest and 6.49 (SD = 0.69) on the post-test. When a regression was performed using a Gaussian distribution, the residuals were not normally distributed; thus, a binomial logistic regression was performed using the median score on the importance of social work partners measure as the median cut point. Neither the main effects of condition nor test were significant (condition: β = 0.80, SE = 0.91, *p* = 0.38; test: β = 0.43, SE = 0.75, *p* = 0.57). Furthermore, the interaction between condition and test was not significant (β = −0.43, SE = 1.04, *p* = 0.68). That is, neither test, condition, nor the interaction between test and condition were associated with beliefs about the importance of social work partners.

### Belief That Pets Affect Owner Health

Participants rated their agreement with statements suggesting that pets positively impact owner health, stress, and physical activity levels, with a score of 7 indicating strong agreement with these statements. Those in the control condition had a mean of 6.83 (SD = 0.29) on the pretest and 6.80 (SD = 0.29) on the post-test. Those in the experimental condition had a mean of 6.64 (SD = 0.47) on the pretest and 6.73 (SD = 0.39) on the post-test. When a regression was performed using a Gaussian distribution, the residuals were not normally distributed, and so a binomial logistic regression was performed using the median score for the belief that pets positively impact health measure as the median cut point. Neither the main effects nor the interaction term were significant (condition: β = −1.02, SE = 0.78, *p* = 0.19; test: β = −0.46, SE = 0.68, *p* = 0.50; condition × test interaction: β = 1.08, SE = 0.96, *p* = 0.26). That is, neither test, condition, nor the interaction between test and condition were associated with beliefs about how pets impact owner health.

### Expectation That Veterinarians Provide Affordable Treatment Options

Participants rated their level of agreement with statements regarding veterinarians' responsibility to provide affordable treatment options, with 1 indicating they strongly disagreed with the statements and 7 indicating they strongly agreed. Participants in the control condition had an average score of 5.87 (SD = 0.85) on the pretest and 6.13 (SD = 0.66) on the post-test. Those in the experimental condition had an average score of 5.69 (SD = 0.74) on the pretest and 6.10 (SD = 0.77) on the post-test. The main effect of condition was not significant (β = −0.18, SE = 0.21, *p* = 0.39), but the main effect of test was (β = 0.26, SE = 0.12, *p* = 0.03). The interaction between condition and test was not significant (β = 0.12, SE = 0.17, *p* = 0.47). Thus, participants showed increases in their expectations that veterinarians provide affordable treatment options from pretest to post-test, regardless of study condition.

### Confidence in Ability to Offer Incremental Care

**Figure 3:**
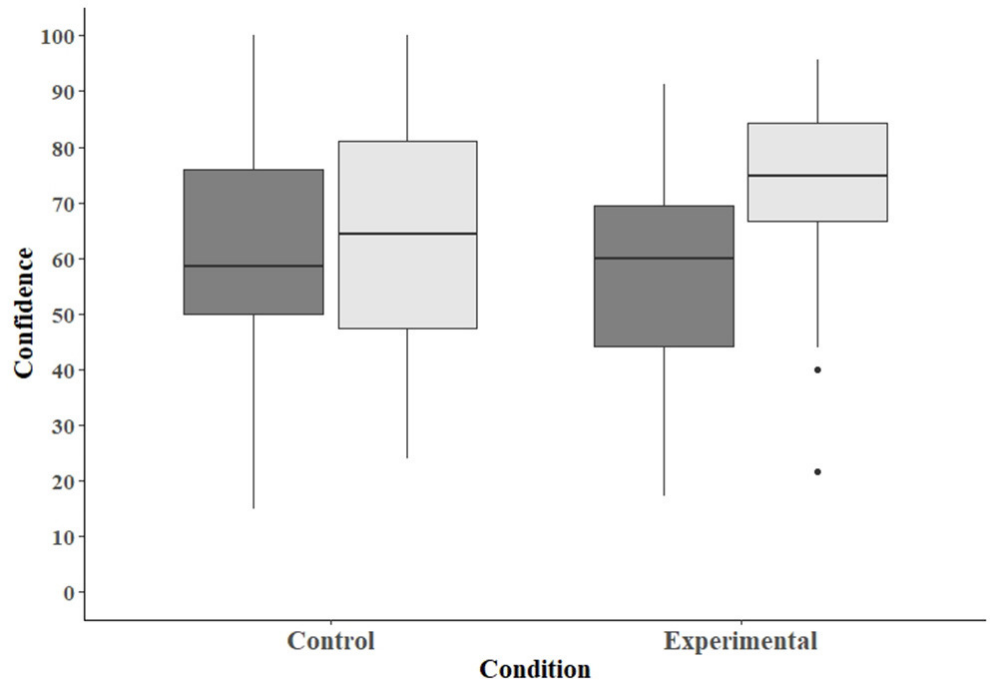
Box plot depicting the scores assessing participants' confidence in their ability to offer incremental care. Dark-gray bars represent scores on the pretest, and light-gray bars scores on the post-test.

Participants could rate their confidence in their ability to provide incremental care on a scale of 0 to 100. The average confidence scores for participants in the control condition were 60.3 (SD = 19.5) on the pretest and 64.2 (SD = 20.9) on the post-test (Figure 3). For individuals in the experimental condition, the average pretest score was 56.2 (SD = 18.4), and the average post-test score was 71.7 (SD = 17.8). The main effects of condition and test were not significant (condition: β =−4.23, SE = 5.23, *p* = 0.42; test: β = 3.87, SE = 3.02, *p* = 0.20); however, the interaction between condition and test was significant (β = 11.60, SE = 4.26, *p* = 0.006). That is, there was a greater increase in confidence scores from pretest to post-test for those in the experimental condition than for those in the control condition.

### Likelihood of Working or Volunteering in Veterinary Desert

Participants were asked to indicate the likelihood they would work or volunteer as a veterinarian in a veterinary desert under a variety of circumstances. Participants were more likely to indicate they would volunteer in a veterinary desert after completing the shelter medicine rotation (β = 1.33, SE = 0.61, *p* = 0.03). There was no effect of experimental condition on the likelihood of volunteering in a veterinary desert (β = 0.27, SE = 0.58, *p* = 0.65), and the interaction between condition and test was not significant (β = −1.33, SE = 0.83, *p* = 0.11). There was no effect of condition or test on the likelihood of working in a for-profit veterinary clinic in a veterinary desert (condition: β = −0.22, SE = 2.06, *p* = 0.92; test: β = 0.78, SE = 1.29, *p* = 0.55), and the interaction between condition and test was not significant (β = −2.31, SE = 2.36, *p* = 0.33). Additionally, there was no effect of condition or test on likelihood of working in a not-for-profit veterinary clinic in a veterinary desert (condition: β = −0.08, SE = 0.75, *p* = 0.91; test: β = 0.87, SE = 0.69, *p* = 0.21), and the interaction between condition and test was not significant (β = −1.59, SE = 1.01, *p* = 0.12). Similarly, the main effects of condition and test and the interaction between condition and test failed to reach significance in relation to likelihood of working in a municipal shelter located in a veterinary desert after graduation (condition: β = −1.10, SE = 0.87, *p* = 0.21; test: β = 0.47, SE = 0.70, *p* = 0.50; interaction: β = −1.32, SE = 1.08, *p* = 0.22).

Participants' impressions of the likelihood that they would be working at a not-for-profit veterinary clinic during the first five years post-graduation were not affected by condition (β = −1.08, SE = 1.03, *p* = 0.29), and the interaction between interaction and test was not significant (β = −0.57, SE = 1.10, *p* = 0.60). The effect of test, however, approached significance (β = 1.44, SE = 0.83, *p* = 0.08). That is, there was a non-significant trend for participants to be more likely to indicate they would work at a not-for-profit veterinary clinic when they took the post-test compared to when they took the pretest, regardless of study condition.

### Responses to Open-Ended Questions

Student responses on the pretest and post-test questions 1 and 2 regarding knowledge on the topic of access to veterinary care indicated that students in both shelter medicine rotations varied widely in terms of their initial familiarity with access to veterinary care issues, and many demonstrated more familiarity after completing the rotation. Both the control and experimental groups' answers were more detailed in 50% of the post-rotation responses to the questions. The following examples are reflective of statements representing differences in students' pre- and post-rotation knowledge:

- **Participant R_6JMJEXumLEL8s2l (Pretest):** “*I do not know much about this at all.”* (rated as minimal familiarity)
- **Participant R_6JMJEXumLEL8s2l (Post-test):** “*During this rotation I have learned more resources for clients who may not have the funds to pay for their pets' medical expenses such as, Scratch Pay, Vet Billing*, & *Vitus Vet Vitus Pay.”* (rated as moderate familiarity)
- **Participant R_1LYfiGtzSybSP7q (Pretest):** “*I know there are some clinics that offer lower cost vet care.”* (rated as minimal familiarity)
- **Participant R_1LYfiGtzSybSP7q (Post-test):** “*There are things like Scratchpay, VetBilling, Vitus VetVitusPay, CareCredit, GoFundMe, fundraisers, and many other options out there to look into for low-income populations. This will allow the pet to get the care they need, give your client more time to pay back the expenses, and your business gets paid and does not risk the client not paying the clinic back. But the limitation is, the client has to have good enough credit to be approved. The second option is that your hospital could partner with the AVMF's veterinary care charitable fund or other charitable foundations. These foundations provide funds to their partnering veterinary clinics so they can offer free or discounted care. This would help the clients that could not afford the care and they were not approved for the credit line. You could partner with a local veterinary school and offer your clinic up for their rotational year. This is a great option because you bring in students who have a good foundation of knowledge but there is no monetary cost to the clinic. This does put a lot of extra stress on the veterinarians and the staff because you are now responsible to help teach and mentor these students while making sure all the work gets done but, it does offer a low cost more hands-on deck option! The last option and the one I would only mention to my most loyal and trusted clients are personalized payment plans with the clinic. However, this is risky and the clinic has to be able to afford it*.” (rated as familiar)

A key word search of the topics or concepts that students mentioned in their responses to the open-ended questions revealed students in the two groups focused on different types of information on the post-test. The control group of students more commonly mentioned economic issues. For instance, 60% mentioned payment plans in the control group vs. 40% in the experimental group, and 100% mentioned pro-bono care in the control group vs. 0% in the experimental group. The experimental group, by contrast, tended to mention more social concepts related to access to veterinary care. For example, they commonly mentioned the existence of veterinary deserts (80% in the experimental group vs. 20% in the control group), implicit bias toward low-income clients (100% in the experimental group vs. 0% in the control group), and incremental treatment plans (75% in the experimental group vs. 35% in the control group). No difference was observed between groups in terms of the prevalence of mentioning the human-animal bond or using grants and subsidies to pay for veterinary care. The examples that follow are reflective of statements representing differences in focus between the two groups of students post-rotation.

- **Participant R_3ssH1WzNZ4Hh6hl (Control Group):** “*More areas than you think are encompassed in low-income care. Overall, they try to do things as low cost as possible. Financial issues are major reasons that animals end up at shelters.”* (coded as cost-of-care)
- **Participant R_ug2aJ1qR0mUQlep (Experimental Group):** “*Just because someone is unable to afford veterinary care currently, doesn't mean that they have been unable to afford it in the past or future. We as practitioners need to learn to recognize implicit bias when it occurs and modify our thinking/actions away from it. Our interaction with our client is a snapshot of that client's life and we have no right to judge what they can/cannot afford or how much they love their animal based on the little information we are provided. Furthermore, a lot of animal surrenders, euthanasia, and untreated animal cases are related to a lack of owner information regarding other options such as pet insurance, outside payment plans, fostering, cheaper procedures at shelters, etc. It is imperative that we as veterinarians open up a dialogue with our clients so that we can provide them with this information which in turn will help our patients.”* (coded as cost-of-care, implicit bias, pet insurance, payment plans, communication skills, incremental care)

Finally, coding of questions 3 and 4, which were asked only to students in the experimental condition, revealed that 32% (*n* = 8) of the respondents desired additional training and resources to use for communicating effectively with clients about access to veterinary care issues. These are some examples of their responses:

- **Participant R_86VVe1bA1LzuHSh:** “*I would have liked to learn how to approach financial conversations with clients. I know this is a skill we will learn in practice but having an example of a difficult conversation would have been helpful for me.”* (coded as communication skills)
- **Participant R_5C2Nzj6n2KzIMHT:** “*I would like to learn more about how to give clients information about alternative payments, euthanasia options, etc. without making them feel like I am judging them or am giving them charity. I personally would also like to learn more about how to start and keep a relationship with the surrounding clinics and shelters.”* (coded as communication skills)
- **Participant R_2ZJ2eJyGnn1bytG:** “*I would like to learn more about what is available in my area. A google search brings up some options but it would be nice if there was a general website that lists known organizations by region.”* (coded as need for additional resources)
- **Participant R_0B96Tyn6pznp7QR):** “*I wish there was a database that is updated with programs in each area of the country. This would make access to them easier for vets who have clients who cannot afford care for their pets.”* (coded as need for additional resources)

## Discussion

Findings from this study demonstrate that veterinary students' knowledge, skills, and attitudes regarding access to veterinary care issues evolved following exposure to content about this topic during a virtual shelter medicine rotation. Some of these changes were observed regardless of whether participants were in the control condition or in the experimental condition that included a specialized, interactive, online learning module that provided in-depth instruction on issues associated with access to veterinary care. At the end of the rotation, participants in both conditions indicated they felt more knowledgeable about the topic of access to veterinary care for low-income and underserved populations. Their opinions about not-for-profit veterinary clinics grew more favorable, and their expectations that veterinarians provide affordable treatment options increased from pretest to post-test. While participants' thoughts regarding whether they would work in a veterinary desert after graduation did not change across the study for either group, participants in both groups were more likely to indicate after the rotation that they would volunteer their professional services in a veterinary desert. Furthermore, there was a non-significant trend suggesting that participants were more likely at the end of the rotation to consider working at a not-for-profit veterinary clinic.

Inclusion of the online learning module for the experimental group did lead to some differences in what students learned about access to veterinary care issues. Students in the experimental group were more likely to describe concepts other than financial factors by the conclusion of the rotation, and they recognized a need for more training on communication skills and for additional easy-to-access web resources. These students were more cognizant of the existence of veterinary deserts, implicit bias, and incremental treatment plans. Furthermore, they showed a reduction in their tendency to be judgmental of veterinary clients from pretest to post-test, and by the end of the study, they expressed greater confidence in their ability to offer incremental care options to veterinary clients. These findings suggest that the online module increased students' understanding of access to veterinary care issues and broadened their mindset beyond traditional “gold standard” of care options. These differences observed between the experimental and control group results might be due to variation between the student learning objectives taught to the two groups, as well as to additional time spent on learning tasks by the students who completed the online module in the experimental condition.

These findings suggest that exposure to the module's content in this virtual shelter medicine rotation may have enhanced students' awareness of how a variety of socio-demographic factors affect the ability of pet owners to access veterinary care. Furthermore, the online module appears to have increased students' confidence in their ability to help pets even when financial resources are constrained. Such knowledge, skills, and attitudes are likely to have favorable impacts on the well-being of pets, their owners, and veterinary staff. That is, putting an access to care perspective into practice may protect companion animals from unnecessary suffering or premature death and enhance the human-animal bond. Additionally, it may buffer veterinarians from stressors associated with discussing the cost of care with clients and performing economic euthanasia. Given the prevalence of burnout, compassion fatigue, and suicide among veterinary professionals (14, 24), any training that leads to a reduction in workplace stressors for those in the veterinary community has the potential to save careers and lives.

Participants' beliefs that pet ownership positively affects health and that partnerships between those in veterinary and social work fields are important did not change from pretest to post-test, regardless of whether participants were in the control or experimental condition. Mean scores on these measures indicate that from the outset of the study, participants held strong, positive beliefs about the relationship between pets and human health and the importance of collaboration between members of veterinary and social work fields. Based on these findings, it seems that veterinary students require little convincing about the importance of these topics, at least at a basic level. Thus, when addressing the human-animal bond and access to veterinary care for poor and underserved individuals, educational modules might focus on concrete ways that veterinarians can support human-animal relationships. For example, they can develop collaborations with social workers and create incremental care plans for clients with limited financial resources.

### Limitations and Future Directions

We found that exposure to content on access to veterinary care clearly resulted in some changes in veterinary students' knowledge and attitudes regarding this topic. While findings from this study are based on self-reported information, which could have introduced social desirability biases, differences observed between the control and experimental groups suggest this bias did not confound study findings. Furthermore, our findings echo those reported by Gongora et al. from their study of cultural competency training opportunities for Australian veterinary students (18). Importantly, participants in our study's control condition did receive some content about access to care and cultural competency as part of the standard shelter medicine rotation offered by Lincoln Memorial University. Removing this standard content from the control condition would have diminished the study's ecological validity. Nevertheless, we still observed that the addition of purposefully curated information about these topics in the experimental condition enhanced student learning in these domains. The differences that were observed between groups may have been due to variation between the study conditions in terms of time spent on learning tasks. Furthermore, it may have been due to the cognitive level at which the learning objectives associated with the access to veterinary care content were presented in the experimental condition as compared to the control condition.

Important questions on this topic remain to be answered. For instance, how long do veterinary students retain the information learned from the online module? Do students' attitudes regarding work with poor and underserved clients persist as they move into their professional careers? Will they have the decision-making ability in their practices to implement strategies that increase access to veterinary care in their communities? Furthermore, as the sample size was relatively small and all students were enrolled at a single institution, the extent to which these findings generalize to other groups of veterinary students is unknown. Further research is needed to determine how long the effects of this training persist and whether these findings are consistent across cohorts of students from a variety of colleges of veterinary medicine. Studies of how this type of training impacts the physical health of companion animals and the mental health of their owners and veterinary staff also are needed.

Additionally, it will be important to evaluate the costs and benefits associated with providing students with an online learning module vs. hands-on opportunities that bring veterinary students face-to-face with the challenges pet owners from underserved communities experience. Indeed, one of the original aims of this study was to make this comparison; however, COVID-19 stymied efforts to provide students with opportunities to participate in wellness clinics in underserved communities. Findings from a qualitative assessment of veterinary students' experiences volunteering at a community veterinary outreach clinic indicate the activity provided opportunities for students to gain more knowledge and acceptance of underserved human populations (25). Likewise, participating in subsidized clinics helps students develop their communication and physical examination skills and feel more comfortable working with clients from underserved communities (26, 27). Comparing the outcomes and efficiencies of classroom-based or online learning modules with those that result from experiential learning opportunities will be necessary to determine what practices are both feasible and effective within contemporary veterinary medical education.

## Conclusions

Access to veterinary care has implications for both companion animal and human health and well-being. The American Medical Association (AMA) includes health equity and access to health care as competencies for medical education and professional ethics (28); however, while strategies and resources exist to improve access to veterinary care in underserved communities, this information typically is not covered in veterinary school curricula in the United States. We found that providing fourth year veterinary students completing a four-week, virtual shelter medicine rotation with an interactive online module, which focused on issues surrounding access to veterinary care, cultural competency, incremental care strategies, and options for payment of veterinary services, increased their awareness of the need to offer a variety of treatment and payment options to veterinary clients. Results from our study can inform curriculum design in veterinary schools and continuing education programs for veterinary professionals.

## Data Availability Statement

The raw data supporting the conclusions of this article will be made available by the authors, without undue reservation.

## Ethics Statement

This study received Institutional Review Board approval from Lincoln Memorial University (IRB #919) and the University of Florida (UF IRB #202001847). Participants provided informed consent electronically.

## Author Contributions

CLH conducted the quantitative analyses and wrote the original draft of the manuscript. TGS conducted qualitative analyses and reviewed and edited the manuscript. KVM secured funding for the study, supervised all aspects of the study, collected the data, and reviewed and edited the manuscript. All authors contributed equally to the conceptualization of the study and subsequent study design.

## Funding

An Intramural Grant awarded to KVM from Lincoln Memorial University, College of Veterinary Medicine provided funding for this project.

## Conflict of Interest

The authors declare that the research was conducted in the absence of any commercial or financial relationships that could be construed as a potential conflict of interest.

## Publisher's Note

All claims expressed in this article are solely those of the authors and do not necessarily represent those of their affiliated organizations, or those of the publisher, the editors and the reviewers. Any product that may be evaluated in this article, or claim that may be made by its manufacturer, is not guaranteed or endorsed by the publisher.
